# Editorial: Zebrafish: An emerging model to study the cellular dynamics of inflammation in development, regeneration, and disease

**DOI:** 10.3389/fcell.2022.1102381

**Published:** 2023-01-09

**Authors:** Con Sullivan, Sofia de Oliveira, Kazu Kikuchi, Benjamin L. King

**Affiliations:** ^1^ College of Arts and Sciences, University of Maine at Augusta, Bangor, ME, United States; ^2^ Albert Einstein College of Medicine, New York, NY, United States; ^3^ National Cerebral and Cardiovascular Center, Suita, Japan; ^4^ Department of Molecular and Biomedical Sciences, University of Maine, Orono, ME, United States

**Keywords:** inflammation, development, regeneration, disease, zebrafish, *Danio rerio*

Inflammation is a complicated process mediated through the multifaceted interactions of innate and adaptive immune cells. Among biomedical model organisms, zebrafish (*Danio rerio*) possess several inherent advantages for studying inflammation, including their small size, relative cost, high fecundity, near transparency, and amenability to genetic manipulation. Numerous fluorescent transgenic lines have been created that enable live imaging of innate and adaptive immune cells including neutrophils (Mathias et al., 2006; Renshaw et al., 2006), macrophages (Ellett et al., 2011), and lymphocytes (Hui et al., 2017; Liu et al., 2017; Ferrero et al., 2020). The zebrafish genome shares over 70% conservation with the human genome, and over 80% of genes associated with human disease have a zebrafish homolog (Howe et al., 2013; Gomes and Mostowy, 2020). Therefore, zebrafish are useful to investigate the regulatory processes of inflammation associated with human diseases. In this Research Topic, we have collected two reviews and three original articles that provide a glimpse into the breadth of research being conducted using zebrafish inflammation models. These articles provide new insights in cardiac and retina regeneration, angiogenesis and hematopoiesis, and disease mechanisms in adolescent idiopathic scoliosis (AIS).

Inflammation is critical for the tissue regenerative response. Grivas et al. and Iribarne and Hyde explored the use of the zebrafish model to untangle the role of new molecular and cellular mediators in heart and retina regeneration, respectively. Grivas et al. provided evidence that zebrafish heart regeneration is inflammation-dependent. In zebrafish, cardiac regeneration requires an initial deposition of scar tissue followed by its degradation. The team explored the role the multifunctional cytokine Mdka plays in the resolution of collagenous scars that arise following cardiac injury and developed a Mdka-deficient zebrafish line (*mdka^cn105^*). The authors found that *mdka^cn105^* zebrafish failed to regenerate cardiac tissue and had more scarring. In addition, *mdka^cn105^* fish had increased levels of collagen and periostin, and TGF-beta signaling, each associated with fibrotic scarring. They also observed that *mdka^cn105^* zebrafish exhibited diminished endothelial cell proliferation, which correlated with reduced expression of *hif1aa* transcripts. Overall, these findings linked the regulation of Mdka expression to the resolution of fibrotic scars and cardiac regeneration. Iribarne and Hyde used acute (NMDA-injured retinas) versus chronic (*gosh* zebrafish mutant) retina injury models to explore the role of inflammation in retinal regeneration. The authors observed a more robust regeneration response in zebrafish exhibiting acute damage than zebrafish exhibiting chronic damage, providing another example for the importance of the inflammatory response in tissue regeneration. NMDA-injured retinas showed a stronger pro-inflammatory response followed by an anti-inflammatory and remodeling response. The retinas of chronically-damage *gosh* zebrafish had a modest pro-inflammatory response with a concomitant, robust anti-inflammatory and remodeling response. It was noted that NMDA-injured retinas had more microglia present than *gosh* mutants, indicating a difference in the inflammatory cellular components involved in the zebrafish response to acute versus chronic damage. Macrophage ablation in both the acute and chronic models caused diminished M1-like microglial activation and regeneration, with less Muller glia proliferation. In contrast, LPS-induced increases in M1-like microglia and proliferating Muller glia were supportive of a retina regeneration response. The authors’ findings demonstrated the effectiveness of these inflammation models in delineating the similarities and differences between acute and chronic retina injuries.

In addition to its role in wound healing and regeneration, inflammation can affect tissue patterning. Subramaniam et al. examined how inflammation influences angiogenesis. Tissue factor (TF) is an inflammation mediator linked to angiogenesis and hemostasis. TF’s role in mammalian vascular development is unclear due to embryonic lethality in mouse knockout models. Therefore, establishment of alternative models to investigate TF-dependent mechanisms is crucial. Subramaniam et al. showed that the zebrafish *f3a* and *f3b* genes are TF gene paralogs. Taking advantage of the morpholino-mediated knockdown approach, the authors demonstrated that *f3a* morphants exhibit an impaired hemostasis phenotype and delayed angiogenesis. Their experiments supported a role for *f3a* in angiogenesis and hemostasis. This zebrafish model will facilitate further inquiries into our understanding of early vascular development, including the role that inflammatory mediators play in this process.

Hematopoiesis is regulated by key inflammatory mediators both in homeostasis and disease (Pietras, 2017). Contrary to zebrafish models, mammalian systems have inherent limitations that restrict visualization and genetic and pharmacological manipulation on a large-scale. Ketharnathan et al. offered a detailed review on how zebrafish models have been used to illustrate the way inflammation influences hematopoiesis, with particular attention given to the influence of inflammation on steady-state hematopoiesis. The authors also discussed the effects of interferon/Jak-Stat signaling on hematopoeisis and blood-related disorders, the linkages connecting ribosomopathies and cytopenias to inflammation, the effect clonal hematopoiesis has on pro- and anti-inflammatory cues, and the role of inflammation in the transition from pre-leukemia to leukemia. The authors highlighted the robustness of the zebrafish models in understanding how inflammation regulates hematopoiesis and contributes to hematopoietic disorders.

Munoz-Montecinos et al. provided a comprehensive review on adolescent idiopathic scoliosis (AIS), which affects up to 4% of the human population and is the most common spinal deformity. The authors described various zebrafish models, with a focus on post-embryonic development, ciliogenesis, cerebrospinal fluid (CSF) flow, and inflammation in AIS. The authors discussed specific inflammatory responses that have been correlated with pathogenesis, including the disruption of CSF dynamics, chronic inflammation, hyperinflammation, increased phosphodiesterase-4 activity leading to elevated NO levels, and increased microglia activity. Munoz-Montecinos et al. noted that there is both localized and systemic inflammation associated with scoliosis. Localized effects include macrophage-driven inflammation along the spinal cord, while the systemic effects can be neuroinflammatory, with increased tumor necrosis factor-alpha levels in the brain. The authors advocated for using phenogenetic approaches in zebrafish models of AIS to gain insights into the cellular and molecular underpinnings that underlie scoliosis phenotypes.

We believe that the articles in the collection demonstrate the potential that zebrafish models have to explore the role of inflammation in developmental, physiological, and disease contexts. The continuous development of and growing interest in zebrafish inflammation models by the research community will provide crucial insights at the interfaces between inflammation and key regulatory mechanisms involved in development, regeneration, and disease processes.
